# Thermal Recovery of Damaged Hydrophobic Coatings in EWOD Devices Using an Integrated Mesh-Patterned Heater

**DOI:** 10.3390/mi17050631

**Published:** 2026-05-21

**Authors:** Youngdoo Son, Woochan Kim, Youngkwang Kim, Daeyoung Lee, Sangkug Chung

**Affiliations:** Department of Mechanical Engineering, Myongji University, Yongin 17058, Republic of Korea; thsduden5@mju.ac.kr (Y.S.); wckim@mju.ac.kr (W.K.); ygkim@mju.ac.kr (Y.K.)

**Keywords:** electrowetting-on-dielectric (EWOD), hydrophobic coating, fluoropolymer, mesh-patterned heater, submersion, falling droplets, durability, reliability

## Abstract

We propose an integrated electrowetting-on-dielectric (EWOD) device incorporating a mesh-patterned heater to restore damaged hydrophobic coatings and evaluate its recovery performance. Hydrophobic degradation was induced under submersion and falling droplet conditions, and the damage and recovery mechanisms of the coating were examined. A damaged Cytop (CTL-809M) coating was thermally treated using the embedded heater at 200 °C for 24 h, successfully restoring its hydrophobicity. Coating properties before and after recovery were characterized by contact angle (CA) and contact angle hysteresis (CAH) measurements, evaluating EWOD performance and surface analyses using atomic force microscopy (AFM) and X-ray photoelectron spectroscopy (XPS). After treatment, the reduced CA and increased CAH were recovered, and wetting/dewetting performance in EWOD operation also recovered to pre-damage levels. AFM and XPS analyses confirmed the simultaneous restoration of the surface morphology and chemical composition. These results demonstrate a practical approach for restoring hydrophobic coatings within EWOD devices and offering a promising solution for improving device reliability and lifetime in applications related to EWOD.

## 1. Introduction

Fluoropolymers are polymers that contain fluorine atoms in their chemical structure. Owing to their outstanding chemical resistance, low surface energy, and low friction, fluoropolymers have been widely used as hydrophobic coatings in a variety of engineering applications. This is due to the unique electronic structure of fluorine atoms, the highly stable C-F bond, and specific intermolecular interactions associated with the organization of fluorinated chains near the surface. Accordingly, the weak interfacial interactions (e.g., van der Waals) between a fluoropolymer and a water droplet lead to a high contact angle and low surface friction, allowing droplets to slide off easily [1,2]. Furthermore, fluoropolymer-based hydrophobic coatings are not limited to imparting water repellency. Their molecular architecture and interfacial bonding can be tailored to the substrate material and surface condition, enabling a range of coating designs optimized for specific applications. These coatings are structurally optimized to achieve adhesion stability, durability, and functional requirements on diverse substrates such as glass, metal, and oxides, and are widely used in fields including chemistry, electrical/electronics, construction, and automotive. In particular, electrowetting-on-dielectric (EWOD) has attracted considerable attention in the micro-electromechanical systems (MEMS) field because of its rapid response time [3,4] and low power consumption [5,6] compared with other droplet manipulation approaches, such as surface acoustic wave (SAW) [7,8,9], mechanical drive [10,11,12], and thermal capillary methods [13,14,15]. Thanks to the properties of fluoropolymers, it has become easier to control droplets in EWOD devices, including droplet transport, vibration, and mixing. These properties enable their use in various applications such as lab-on-a-chip [16,17,18], liquid lens [19,20,21], electrowetting display (EWD) [22,23,24], and droplet-based energy generator [25,26]. Beyond EWOD-based systems, hydrophobic coatings are also widely utilized in other lab-on-a-chip platforms, such as dielectrophoresis and optoelectronic tweezers, as well as in non-droplet-based particle manipulation systems, where controlled interfacial properties are essential [27,28,29].

However, the hydrophobic top coating, a key component in EWOD devices, has been reported to have hydrophobicity degradation under various factors, including droplet oscillation on the surface [30], mechanical abrasion [31,32], repeated electrowetting actuation [33], UV exposure [34,35], and prolonged contact with water [30,36]. To overcome this limitation, several studies have investigated recovery strategies for damaged hydrophobic layers. Gnanappa et al. measured the contact angle of a fluorocarbon coating during 12 days of water immersion and observed a continuous decrease, which was subsequently restored by annealing at 100 °C for 1 h. Furthermore, the damage and recovery mechanisms were analyzed simultaneously using atomic force microscopy (AFM) and X-ray photoelectron spectroscopy (XPS) [37]. Trapuzzano et al. similarly degraded multiple fluoropolymer coatings commonly used in industrial EWOD devices via long-term submersion and demonstrated that thermal treatment at 160 °C could recover hydrophobicity for certain coatings [30].

Nevertheless, most previous studies have primarily focused on demonstrating the thermal recovery of fluoropolymer coatings themselves, while the recovery of hydrophobic coatings within an integrated EWOD device structure has been less explored. In practical EWOD systems, global heating of the entire device may expose not only the degraded hydrophobic coating but also surrounding functional layers and electronic components to unnecessary thermal stress. Such thermal exposure may cause physical and chemical degradation of temperature-sensitive materials and surrounding electronic components [36,38,39]. Therefore, an in-device localized heating strategy can provide a more device-compatible approach by applying thermal energy near the degraded hydrophobic coating region, while potentially reducing thermal exposure to surrounding components. In this context, this study proposes an EWOD device incorporating a mesh-patterned heater as a proof-of-concept platform for localized thermal recovery of damaged hydrophobic coatings.

**Figure 1:**
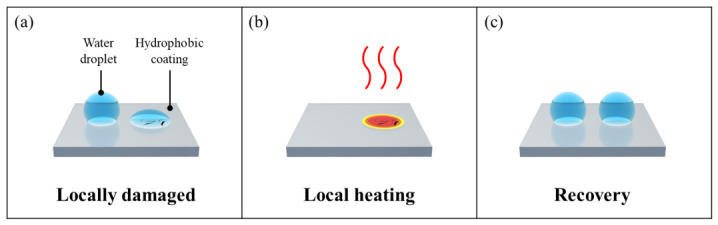
Schematic diagram of (**a**) locally damaged hydrophobic coating; (**b**) local heating to recover locally damaged hydrophobic coating; (**c**) recovered hydrophobic coating.

Figure 1 illustrates the overall concept of this study. Cytop (CTL-809M) was used as the hydrophobic coating. The M-type hydrophobic coating from Cytop is widely used in EWOD applications and offers the advantage of excellent adhesion stability through covalent bonding with ${SiO}_{2}$ substrates. In order to evaluate the damage and recovery mechanisms of such hydrophobic coatings, we selected submersion and falling droplet as the two damage modes in this study. Submersion was chosen to consider only the simple interaction with water, while droplet impact was selected to add the complex effect of repeated physical impacts from falling water droplets as well as the interaction with water. This dual approach aims to verify whether recovery using heating is effective for the two methods of damaging the hydrophobic coating. Through these two methods, the damaged hydrophobic coating (Figure 1a) is subsequently heated at a constant temperature for 24 h via the embedded mesh-patterned heater, restoring its hydrophobicity. (Figure 1b,c).

Previous studies evaluated only the contact angle (CA) and contact angle hysteresis (CAH) of droplets placed on the hydrophobic coating to determine whether it recovered through heating. Additionally, this study analyzed the EWOD performance to verify the recovery effect more quantitatively. Furthermore, changes in surface morphology and chemical composition due to the damage and recovery of the hydrophobic coating were observed using AFM and XPS, respectively. These observations enabled an in-depth analysis of the damage and recovery mechanisms of the hydrophobic coating.

## 2. Principles

### 2.1. Electrowetting-on-Dielectric

**Figure 2:**
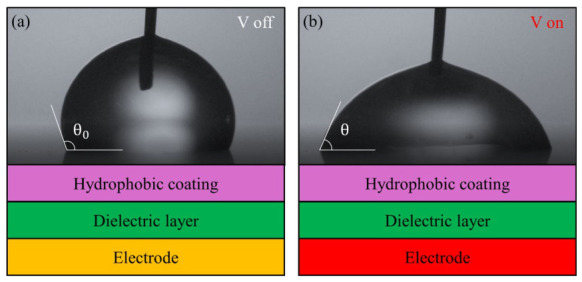
Wettability changes in electrowetting-on-dielectric (EWOD): (**a**) initial state and (**b**) after voltage is applied between a conducting droplet and an electrode.

The theoretical foundation of electrowetting on dielectric (EWOD) was established in the early 2000s [4,40,41]. The Lippmann-Young equation, which describes the relationship between voltage and the contact angle (CA) of a conductive droplet, plays a crucial role in all major physical principles of EWOD. When a voltage is applied between a conductive droplet and an electrode coated with a dielectric layer, as shown in Figure 2, an electric double layer spontaneously forms near the solid–liquid interface of the dielectric layer. Under an electric field, charge accumulation around the three-phase contact line (TCL) reduces interfacial tension, causing the droplet to spread. Berge et al. [40] derived the following equation by substituting the Lippmann equation, which describes the relationship between applied voltage and droplet contact angle, into the Young equation, which describes the relationship between the interface and the droplet contact angle.

(1) $$\cos\theta =\cos\theta_{0}+\frac{\varepsilon_{0}}{2\gamma }\left(\frac{\varepsilon_{D}\varepsilon_{H}}{\varepsilon_{D}d_{H}+\varepsilon_{H}d_{D}}\right)V^{2}$$

Here, θ denotes the contact angle at an applied potential V, and $\theta_{0}$ is the initial contact angle at zero potential. $\varepsilon_{0}$ is the vacuum permittivity, $\varepsilon_{D}$ is the relative permittivity of the dielectric layer, and $\varepsilon_{H}$ is the relative permittivity of the hydrophobic layer. γ represents the interfacial tension between the droplet and the surrounding fluid. $d_{D}$ and $d_{H}$ are the thicknesses of the dielectric layer and the hydrophobic layer, respectively. This equation indicates that the droplet contact angle decreases as the applied voltage increases.

### 2.2. Joule Heating

When an electrical signal is applied to a conductive material with resistance, according to Joule’s law, the material generates heat proportional to the power. This generated heat is dissipated through conduction, convection, and radiation, eventually reaching an equilibrium temperature [42]. This process can be expressed by Equation (2) as follows:

(2) $$I^{2}R=\left(m_{1}C_{1}+m_{2}C_{2}\right)\frac{dT(t)}{dt}+A\left(h_{1}+h_{2}\right)\left(T\left(t\right)-T_{0}\right)+\sigma A(\varepsilon_{1}+\varepsilon_{2})({T(t)}^{4}-T_{0}^{4})$$

First, the left-hand side of the equation represents the electrical power, where I is the current and R is the electrical resistance. The expression on the right-hand side of the equation represents the heat dissipation process, where the first term corresponds to conduction, the second term to convection, and the last term to radiation. Common variables include the substrate area A, the temperature T(t) at time t, and the ambient temperature $T_{0}$. In each parameter, the indexes 1 and 2 denote the electrode and substrate for heat generation, respectively. In the first term for conduction, m denotes the mass and C denotes the specific heat capacity. In the second term for convection, h denotes the convective heat-transfer coefficient. In the final term for radiation, σ is the Stefan–Boltzmann constant and ε is the emissivity. Consequently, both the thermal response time and the steady-state equilibrium temperature of the device can be precisely controlled by modulating the applied voltage [43,44].

## 3. Design and Fabrication

**Figure 3:**
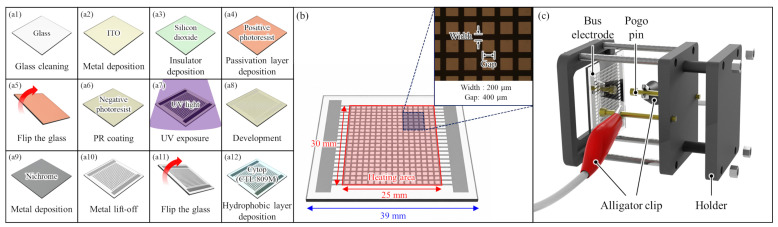
Schematic diagram of fabrication of mesh-patterned heater for recovery of hydrophobic coating in EWOD devices: (**a1**–**a12**) fabrication process, (**b**) specifications of the mesh-patterned heater and (**c**) illustration of operating mechanism.

The samples were fabricated using a 1.1 mm thick borosilicate glass substrate selected for its high thermal stability. The top side of the substrate was configured with the EWOD structure, while the opposite side incorporated a mesh-patterned heater. The fabrication sequence was determined by considering the material properties of each thin layer and overall process compatibility, as shown in Figure 3(a1–a12). The fabricated mesh-patterned heater operates based on Joule heating. As illustrated in Figure 3b, a DC voltage was applied using pogo pins connected via alligator clips to the bus electrodes on both sides of the heater.

In order to form electrodes for the EWOD structure, a 100 nm thick indium tin oxide (ITO) was deposited by sputtering onto the top surface of a 39 mm × 39 mm borosilicate glass substrate. Subsequently, a 1 μm thick ${SiO}_{2}$ dielectric layer was then deposited using plasma-enhanced chemical vapor deposition (PECVD). Since the ${SiO}_{2}$ surface is vulnerable to mechanical impact or scratches, which could cause insulation breakdown during subsequent EWOD operation [45,46]. To prevent this, a positive photoresist (AZ-GXR 601) was spin-coated onto the ${SiO}_{2}$ surface prior to fabricating the mesh-patterned heater. It was then hard-baked at 120 °C for 10 min to serve as a temporary protective layer.

The mesh-patterned heater on the backside was fabricated by a lift-off process using nichrome. Nichrome was selected for its high electrical resistivity and oxidation resistance, enabling stable heat generation under prolonged high-temperature operation without significant resistance changes or degradation. For heater patterning, a negative photoresist (AZ-nLOF 2035) was spin-coated and patterned on the backside glass surface. A 100 nm thick nichrome layer was then deposited by electron-beam evaporation, followed by lift-off in acetone and rinsing in isopropyl alcohol. The substrate was cleaned with deionized water and dried with nitrogen gas to complete the mesh heater. Finally, a 250 nm thick Cytop (CTL-809M) layer was spin-coated on top of the ${SiO}_{2}$ and baked according to the manufacturer’s manual.

## 4. Experimental Setup

### 4.1. Damage

**Figure 4:**
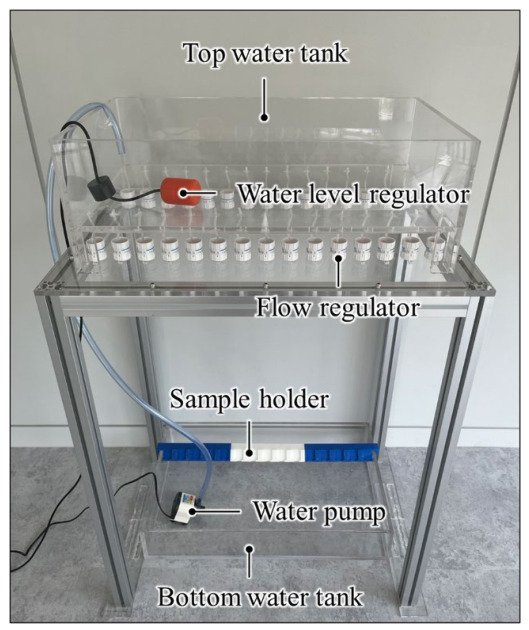
Experimental setup for damaging the hydrophobic coating by submersion and falling droplet.

An experimental setup was designed to simultaneously damage multiple EWOD samples via submersion and falling droplet, as shown in Figure 4. The 3D-printed holder could stably hold fifteen samples at once. This holder was designed to tilt at a 45-degree angle relative to the ground, allowing water to effectively slip off the sample surface and thereby minimizing the effects of unintended submersion. Furthermore, the holder could be easily mounted at a fixed position on an acrylic water tank, enabling simple sample attachment and removal while ensuring experiments were always conducted at the same location.

For the falling droplet method, the top water tank was positioned 800 mm above the holder. Using a flow regulator, water droplets of approximately 70 μL were dropped onto the center of each sample at a rate of one drop per second. When the water level in the top water tank fell below a preset threshold, a water level regulator activated a water pump in the bottom tank to maintain a constant water level in the top tank. This setup enabled the stable reproduction of repetitive droplet impacts.

In contrast, the submersion test was performed by simply submerging the sample completely in the bottom water tank using a standard sample holder. This simulated a different type of degradation behavior distinct from that caused by falling droplets. Under these two damage conditions, we were able to evaluate the degradation characteristics of the hydrophobic coating under various environmental conditions. This also enhanced the reproducibility and comparability of data following recovery experiments.

### 4.2. Recovery

**Figure 5:**
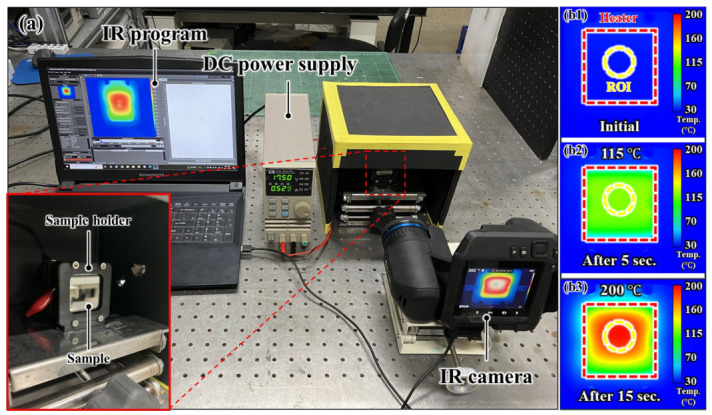
(**a**) Experimental setup to recover the damaged hydrophobic coating with mesh-patterned heater, (**b1**–**b3**) temperature measurements in the ROI (region of interest) using an infrared camera.

A setup, as shown in Figure 5, was devised to consistently heat the damaged sample to a constant temperature using mesh-patterned heater. The damaged sample was fixed using a holder fabricated via a resin 3D printer, and a DC power supply was used to apply a drive signal to the mesh-patterned heater. An infrared camera was used to observe the temperature distribution and maximum temperature of the sample, and temperature measurements were taken at the center of the sample, which was the region of interest in this study. The voltage and current of the DC power supply were determined based on the measured temperature. To prevent measurement errors caused by light scattering or reflection during IR imaging, the ambient light around the sample was blocked.

### 4.3. Contact Angle and Contact Angle Hysteresis Measurements

**Figure 6:**
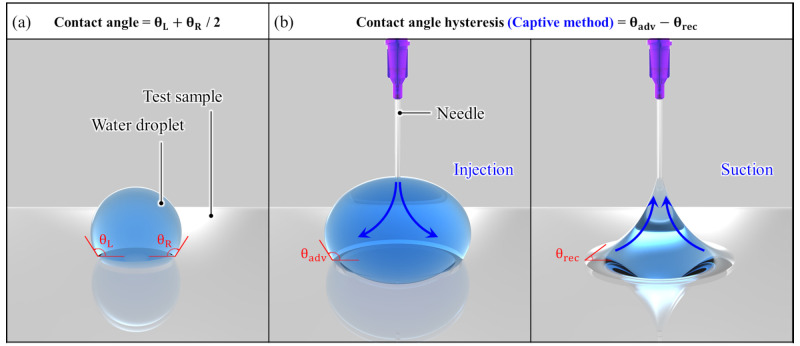
Experimental setup for measuring (**a**) contact angle and (**b**) contact angle hysteresis.

To evaluate the hydrophobicity of the samples, the CA and CAH of a water droplet placed on the surface were measured using a contact angle goniometer (Smartdrop, Femtobiomed Inc., Seongnam-si, Republic of Korea), as illustrated in Figure 6. The CA was determined by dispensing a 5 μL droplet onto the sample surface. The contact angles at the left and right three-phase contact lines were automatically calculated using the image analysis software and then averaged. The CAH of each sample was measured using the captive method. First, a 5 μL droplet was placed on the sample surface, and the droplet volume was precisely controlled using a syringe integrated with the Smartdrop system. The droplet volume was then increased by dispensing water, which expanded the contact area between the droplet and the sample surface. The contact angle measured during this process was defined as the advancing contact angle ($\theta_{adv}$). Subsequently, the same droplet was used for the receding measurement by withdrawing water through the syringe. As the droplet volume decreased, the contact area between the droplet and the sample surface decreased, and the corresponding contact angle was defined as the receding contact angle ($\theta_{rec}$). The CAH was calculated as the difference between $\theta_{adv}$ and $\theta_{rec}$. The droplet volume change during the captive method was automatically controlled by the Smartdrop software. Each sample was measured at least three times under identical conditions, and the results were obtained as the mean value and standard deviation.

## 5. Results and Discussion

**Figure 7:**
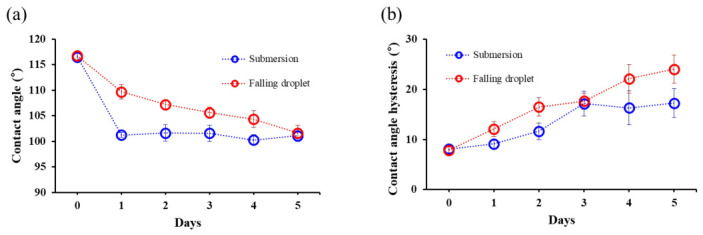
Changes in the hydrophobic coating during damage: (**a**) contact angle and (**b**) contact angle hysteresis.

Each sample was damaged for 5 days using submersion and falling droplet methods, with CA and CAH measured on a daily basis, as shown in Figure 7. The results showed that the CA decrease was approximately 15° for both methods, but the submersion samples reached this value in a single day, whereas the falling droplet samples took 5 days. In contrast, the CAH increased at a rate of 1.8° per day for the submersion samples, but increased at a rate of 3.2° per day for the falling droplet samples, resulting in a 6.8° greater CAH for the falling droplet samples. Subsequently, the damaged samples were kept at room temperature for one month. As a result, regardless of the damage method, the CA decreased to approximately 75°, while the CAH increased to 27.6° for the submersion samples and 32.5° for the falling droplet samples.

**Figure 8:**
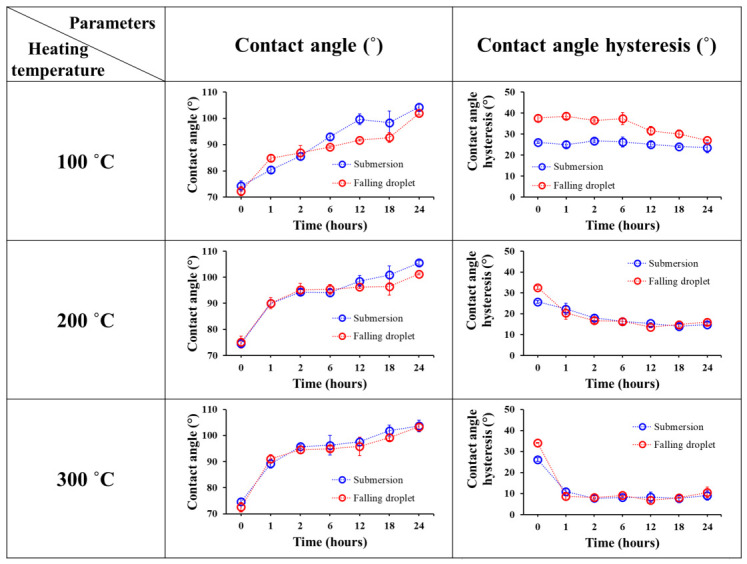
Changes in contact angle and contact angle hysteresis of the hydrophobic coating as a function of heating time under different temperatures.

To determine the optimal heating temperature for damaged samples, they were heated at 100 °C, 200 °C, and 300 °C for 24 h as shown in Figure 8. Higher heating temperatures generally resulted in greater and faster recovery of CAH, whereas the CA recovery rate did not show significant differences. After heating at 300 °C, the final recovered CAH reached 9.8°, representing the highest recovery among the tested temperatures. Although heating at 300 °C resulted in faster CAH recovery, the CA recovery required approximately 24 h regardless of the heating temperature. Therefore, considering the risk of thermal-stress-induced fracture, 200 °C was selected as the recovery temperature in this study. Nevertheless, 300 °C may still be a viable option for applications in which the substrate can tolerate higher-temperature treatment.

**Figure 9:**
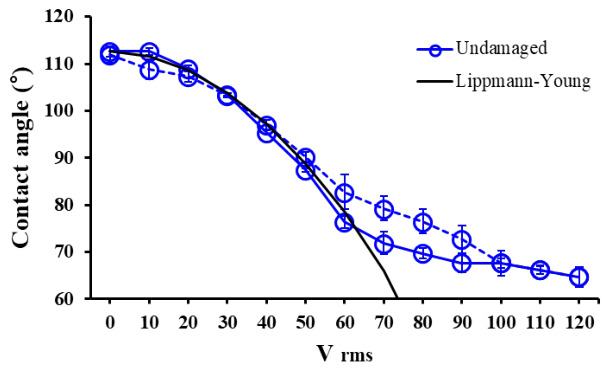
EWOD hysteresis graph of undamaged samples.

Wetting and dewetting performance are critical factors determining the efficiency of EWOD devices. To enhance this performance, the wetting phenomenon—where the contact angle of the water droplet decreases with applied voltage—must be maximized. Additionally, when the applied voltage is removed, the water droplet must dewet and recover to a contact angle close to its initial value. Thus, EWOD hysteresis, defined as the difference between the initial contact angle and the contact angle measured after the applied voltage is increased during the EWOD test and then sequentially decreased back to 0 V, was evaluated as an indicator of EWOD device performance (Figure 9) [47,48].

Figure 9 shows the EWOD hysteresis curve of undamaged samples. The EWOD actuation tests were conducted using a function generator (33210A, Agilent Co., Santa Clara, CA, USA) and an amplifier (PZD700, Trek Co., Medina, NY, USA). The voltage was sequentially increased from 0 V_rms_ to 120 V_rms_ in 10 V_rms_ increments at a frequency of 1kHz, then decreased back to 0 V_rms_ in the same manner to measure the CA change of the water droplet. The undamaged sample closely followed the theoretical Lippmann–Young model. The difference from the theoretical value above 70 V is due to contact angle saturation [49,50]. The undamaged sample showed a contact angle of approximately 65° at 120 V_rms_ and showed approximately 1° of EWOD hysteresis after voltage removal.

**Figure 10:**
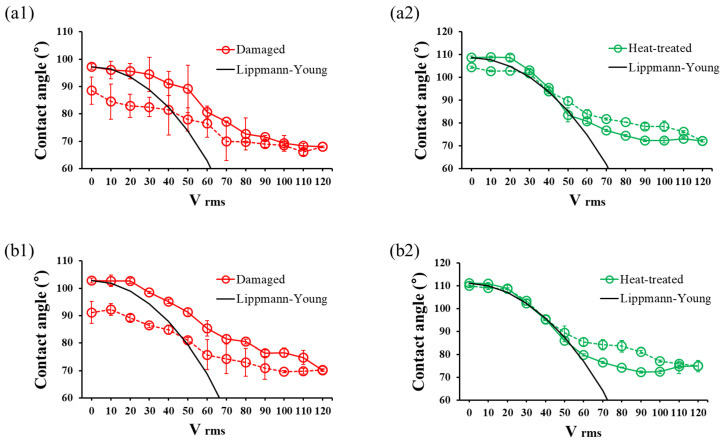
EWOD hysteresis curves as a function of applied voltage for (**a1**,**a2**) submersion and (**b1**,**b2**) falling droplet samples. The left and right columns indicate damaged and heat-treated samples. The solid line is for increasing voltage, and the dashed line is for decreasing voltage.

Subsequently, the EWOD performance of samples damaged by submersion and falling droplets was compared. Figure 10 presents the EWOD hysteresis curves for each condition. Both damaged samples deviated from the Lippmann-Young theoretical value. Furthermore, when the voltage was sequentially applied up to 120V and then reduced, dewetting did not occur effectively due to the increased CAH after damage. As a result, the submersion and falling droplet samples showed EWOD hysteresis of approximately 7° and 11°, respectively.

However, after heating at 200 °C for 24 h using an embedded mesh-patterned heater, the EWOD hysteresis graph aligned well with theoretical values, showing effective dewetting. As a result, the samples damaged by submersion and falling droplets recovered to approximately 4° and 1°, respectively.

**Figure 11:**
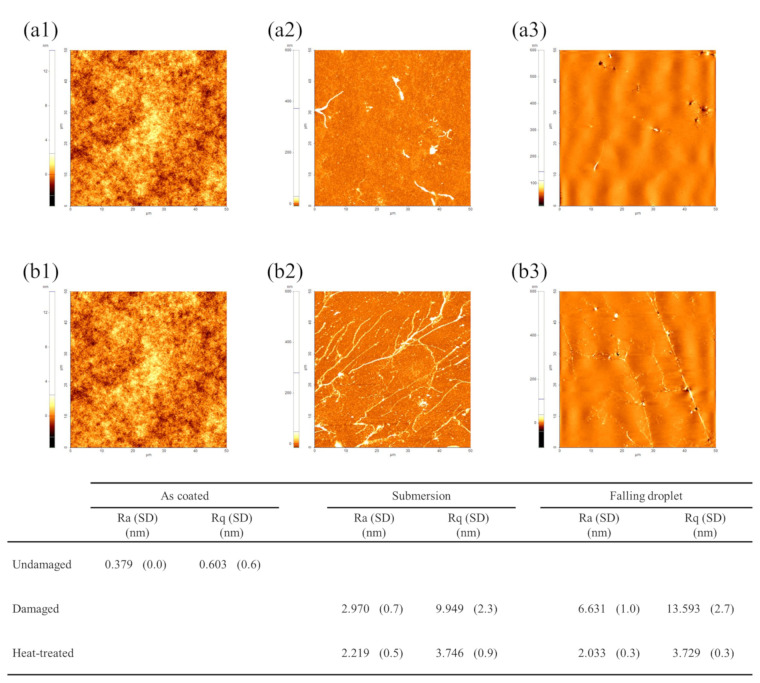
Atomic force microscopy images and corresponding surface roughness values of hydrophobic coatings: (**a1**–**a3**) submersion samples and (**b1**–**b3**) falling droplet samples. From left to right: undamaged, damaged, and heat-treated states. The table summarizes the average roughness (Ra) and root-mean-square roughness (Rq) with standard deviation (SD).

To analyze the damage and recovery mechanisms of the damaged hydrophobic coating by each method, surface morphology was observed using AFM (XE-100, PSIA Corp., Seongnam-si, Republic of Korea). Each sample was measured within an area of 50 μm × 50 μm, the maximum measurement range of the equipment. Figure 11 shows the AFM images of the hydrophobic coating before damage, after damage, and after recovery. The sample before damage showed very low roughness, indicating a uniform surface. In contrast, after damage, ridge-like protruding structures formed on the surface, causing a significant increase in surface roughness.

The formation of ridge structures is considered to be related to the molecular structural characteristics of Cytop (CTL-809M) and its exposure to water. The M-type Cytop hydrophobic coating consists of an interfacial anchoring layer covalently bonded to the substrate via silane functional groups, while the upper region is composed of fluorinated polymer chains, providing relatively high chain mobility near the surface [51]. This structural feature suggests that the near-surface region can undergo segmental rearrangement or localized structural changes in response to external environmental conditions.

Based on the AFM observations, two possible mechanisms can be considered. One possibility is that, during water exposure, moisture locally penetrates through microdefects or free volume regions within the polymer, leading to partial swelling. Subsequent non-uniform shrinkage during drying or measurement may result in the formation of protruding ridge-like structures [52,53]. Another possibility is that interaction with water induces non-uniform reorientation of polymer chains near the surface, generating localized surface stress [54]. To relieve this stress, localized buckling or wrinkling in the form of ridge structures may occur.

Experimentally, these ridges were observed more frequently in the falling droplet samples, where the surface roughness increased by approximately six times compared to the submersion samples. This difference is likely associated with the combined effects of repeated mechanical impact and water exposure in the falling droplet condition.

After thermal recovery, the ridge structures were significantly reduced, and the surface morphology became more uniform. The roughness of both samples also returned to similar levels. These results indicate that the increase in CAH after damage is closely associated with the increase in surface roughness. Furthermore, the recovery behavior suggests that thermal treatment facilitates structural relaxation and reorganization of the near-surface polymer chains, leading to the restoration of a smoother surface morphology and improved hydrophobic performance.

**Figure 12:**
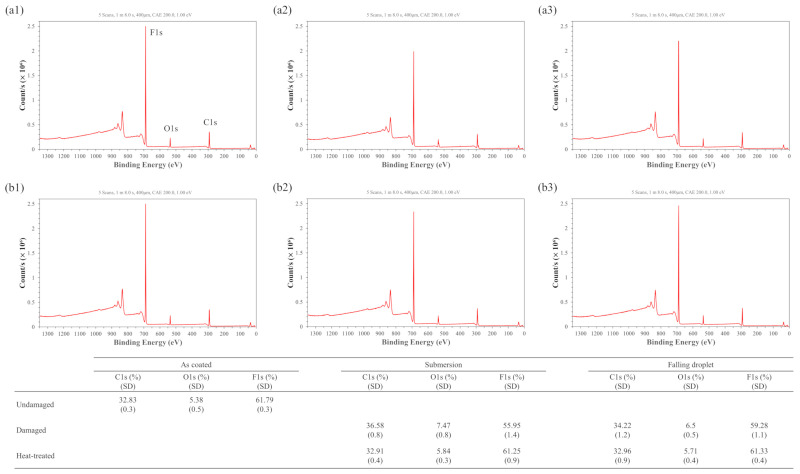
X-ray photoelectron spectroscopy spectra and elemental composition of hydrophobic coatings: (**a1**–**a3**) submersion samples and (**b1**–**b3**) falling droplet samples. From left to right: undamaged, damaged, and heat-treated states. The table summarizes the atomic percentages of C1s, O1s, and F1s with standard deviation (SD).

Figure 12 shows the XPS (Nexsa, Thermo Fisher Scientific, Waltham, MA, USA) results of the undamaged, damaged, and heat-treated samples. Measurements were taken over a 400 μm × 400 μm area centered on the sample, and the surface ratios of carbon, oxygen, and fluorine were analyzed. After damage, the fluorine ratio at the surface of the hydrophobic coating decreased, whereas the carbon and oxygen ratios increased. This trend suggests that water exposure partially reduced the surface contribution of fluorinated polymer segments, while oxygen-containing components and/or adsorbed water became relatively more dominant near the surface. Therefore, the observed compositional change is interpreted as a redistribution or change in the relative surface exposure of near-surface components rather than direct evidence of the formation of new chemical species. As a result, the surface energy increased, leading to a decrease in CA.

Notably, these compositional changes were more pronounced in the submersion samples than in the falling droplet samples. This is likely because, under the submersion condition, the fluorinated polymer surface remained in continuous contact with water for a longer period, allowing interfacial hydration and water-induced surface reorganization to proceed more extensively. Consequently, the fluorine signal was more strongly reduced and the relative contribution of oxygen-containing components increased, which explains the more rapid decrease in CA compared with the falling droplet samples.

After heat treatment, the fluorine ratio increased again, while the carbon and oxygen ratios decreased. This indicates that the surface recovered toward a fluorine-rich, low-surface-energy state. This recovery is likely associated with thermally induced chain mobility, which allows fluorinated polymer segments to reorient toward the air interface and reduces the relative exposure of oxygen-containing components, thereby restoring the hydrophobic surface state [55].

Overall, the decrease in CA after hydrophobic coating damage is primarily associated with the surface compositional changes observed by XPS, particularly the reduced surface contribution of fluorinated segments and the increased relative contribution of oxygen-containing components. In contrast, the increase in CAH is closely related to the ridge structures observed by AFM and the resulting increase in surface roughness, which was more pronounced under falling droplet conditions. Therefore, the simultaneous improvement in CA and CAH after heating can be interpreted as the combined recovery of surface chemical composition and surface morphology.

## 6. Conclusions

This study presents an integrated EWOD device incorporating a mesh-patterned heater to restore damaged hydrophobic coatings and evaluates its performance. To optimize the heating temperature, samples were treated at 100 °C, 200 °C, and 300 °C for 24 h. The results indicated that 200 °C was the optimal temperature for achieving stable recovery of both CA and CAH while minimizing potential thermal stress on the substrate.

Hydrophobic degradation was induced under two conditions, submersion and falling droplets, both of which resulted in the typical phenomena of decreased CA and increased CAH. For submersion samples, XPS revealed a decrease in the fluorine ratio and an increase in carbon and oxygen ratios, indicating that changes in surface chemical composition primarily contributed to the reduction in hydrophobicity. In contrast, under the falling droplet condition, AFM observations revealed a significant increase in surface roughness and the formation of ridge structures, suggesting that repeated physical impacts and the resulting surface morphology changes played a dominant role. These findings underscore that hydrophobic degradation is not governed by a single factor but arises from a complex interaction between chemical composition and surface morphology, depending on the environmental exposure.

After 24-h heating at 200 °C using an embedded mesh-patterned heater, the subsequent EWOD performance showed that the EWOD hysteresis, which had increased due to damage, was effectively reduced. This restored the wetting and dewetting performance to nearly pre-damage levels. AFM analysis showed that the ridge structures formed during damage and the resulting increase in roughness were significantly mitigated after heating, verifying that the surface recovered to a uniform state. Furthermore, XPS analysis showed that the surface exposure of fluorine, which had decreased after damage, was restored after heating, while carbon and oxygen decreased. These results suggest that both surface morphology and chemical composition were partially restored after thermal treatment. This recovery may be associated with thermally induced relaxation and reorganization of near-surface polymer chains, leading to a lower surface energy state.

Overall, these results demonstrate that in situ localized heating via an embedded mesh-patterned heater can effectively restore damaged hydrophobic coatings within EWOD devices, thereby ensuring device reliability and long-term durability. This research is expected to contribute to enhancing both the stability and lifespan of EWOD-based applications.

## Data Availability

The original contributions presented in this study are included in the article. Further inquiries can be directed to the corresponding authors.

## Abbreviations

The following abbreviations are used in this manuscript:

EWOD	Electrowetting-on-dielectric
MEMS	Micro-electromechanical systems
CA	Contact angle
CAH	Contact angle hysteresis
AFM	Atomic force microscopy
XPS	X-ray photoelectron spectroscopy
